# Effect of Au Nanoparticle Agglomeration on SERS Signal Amplification

**DOI:** 10.3390/nano13050812

**Published:** 2023-02-22

**Authors:** Kirill Khabarov, Emiliia Filalova, Messan Nouraldeen, Ekaterina Kameneva, Andrey Musaev, Sergei Tikhonov, Victor Ivanov

**Affiliations:** Moscow Institute of Physics and Technology, National Research University, 9 Institutskiy per., Dolgoprudny, 141701 Moscow, Russia

**Keywords:** nanoparticles, gold, Raman spectroscopy, surface-enhance Raman scattering, plasmonics

## Abstract

An analyzed substance’s signal intensity and detection sensitivity in surface-enhanced Raman spectroscopy (SERS) significantly depend on the size and agglomeration degree of nanoparticles (NPs) forming the enhancing structure. Structures were manufactured by aerosol dry printing (ADP), where NPs’ agglomeration depends on printing conditions and additional particle modification techniques. The influence of agglomeration degree on SERS signal enhancement was studied in three types of printed structures using the methylene blue model molecule as an analyte. We demonstrated that the ratio between individual NPs and agglomerates in a studied structure strongly affects SERS signal amplification, and structures formed mainly from non-agglomerated NPs enhance the signal better. In this sense, aerosol NPs modified by pulsed laser radiation provide better results than thermally modified NPs, since in laser modification a larger number of individual NPs is observed due to the absence of secondary agglomeration effects in the gas stream. However, increasing gas flow may minimize the secondary agglomeration, since the time allotted for the agglomeration processes is reduced. In this paper, we show how different NPs’ agglomeration tendencies influence SERS enhancement to demonstrate the process of using ADP to form inexpensive and highly efficient SERS substrates with huge application potential.

## 1. Introduction

At present, various methods for detecting and characterizing organic substances exist [1,2,3,4,5]. Among them, SERS is one of the most advanced and most sensitive nondestructive express techniques [6,7]. It finds application in environmental monitoring tasks [8], biomedical diagnostics [9], detection of trace impurities in substances [10], and other applications. In this method, the signal is inelastically scattered by the analyzed substances (analytes), amplified by the surrounding nanostructure, and detected in the far-infrared (IR) range, where it describes the vibrational states of chemical bonds characteristic of a studied analyte molecule [6]. The nanostructure’s properties, such as its material and morphology, are carefully selected to maintain the strong localization of the electromagnetic field, enhance the analyte response, and determine the method’s sensitivity overall. For instance, ordered and disordered arrays of nanospheres [11,12,13], nanodiscs [14], nanostars [15], and other nanostructured patterns [16] made of noble metals (silver, gold, copper), transition metals [17], as well as dielectrics and semiconductor materials [18] can be used for these purposes.

Lithography and deposition from colloidal solutions [19] are the main methods of forming SERS structures based on nanoobjects. These methods are complex and inevitably use chemicals that reduce the purity of the studied analyte, for example, photoresist, surfactants, and dispersants. In that sense, magnetron sputtering technology and physical deposition methods [20] are able to form pure and high-qualitative SERS substrates; however, the production of these samples requires more time and resources because the deposition of the material is carried out on the entire available surface in the setups. ADP based on dry NPs [21] is devoid of these disadvantages and represents an alternative approach to the formation of SERS substrates. The major advantage of the method is the contactless NPs deposition in real time that can be produced on various surfaces. This process allows depositing NPs arrays on top of an analyzed substance to study its SERS signal [22] and keeps the analyte intact, which is particularly suitable for studying cultural heritage sites [23]. It is convenient to synthesize NPs for this technology with a spark discharge [24], which can be easily controlled with a fast response due to the instantaneous change in the electrical parameters of the discharge circuit. An extensive set of spark discharge parameters also makes the fine control of the size and concentration of synthesized NPs possible. Additionally, the size, concentration, and shape of aerosol NPs may be modified by an external influence of high temperatures [25] or laser radiation [26]. However, the high tendency of NPs to agglomerate during their transportation in ADP results in a reduction in the plasmonic properties of the printed sample in the visible wavelength range [27], which decreases the efficiency of its usage in SERS. Therefore, a need exists to understand how arrays formed in approaches with different NPs agglomeration tendencies can influence SERS.

In this paper, we demonstrate the significant contribution of NP agglomeration that takes place in ADP in order to improve the properties of printed structures to achieve greater SERS signal amplification. We verified this by the investigation of the SERS signal amplification efficiency and theoretical limits of analyte detection for SERS structures on the example of the methylene blue (MB) model molecule. The structures were formed by the ADP of gold (Au) NPs on aluminum oxide (Al_2_O_3_) substrates. The samples were formed in one layer in three ways: from agglomerates of primary NPs with an average size of about 10 nm, from NPs obtained by heating initial agglomerates to 750 °C, and from NPs obtained from initial agglomerates exposed to nanosecond pulsed laser radiation with a wavelength of 1053 nm. We also changed the initial agglomerates’ average size and concentration by varying the gas flow in the experiments.

## 2. Materials and Methods

### 2.1. Aerosol Dry Printing of SERS Structures

SERS structures were fabricated from Au NPs deposited by the aerosol dry printer in a single layer on aluminum-oxide substrates in square arrays with a side of 3 mm. NPs were synthesized with spark discharge during the electrical erosion of gold electrodes in an atmosphere of high-purity argon (99.9999%). Gold electrodes in the form of hollow cylinders with outer and inner diameters of 8 and 6 mm, respectively, were used. The purity of the electrodes’ material was 99.9999%.

Primary NPs with an average size of about 10 nm were synthesized in three experiments with gas flow rates Q = 50, 200, and 400 mL/min. During the movement of aerosol flow, due to the Brownian motion, NPs experience many collisions and combine into arbitrarily shaped dendritic agglomerates with an average size of 150–300 nm. It is important to note that at lower flow rates, the size of agglomerates was larger because the time of NPs being in the flow increased. Additionally, the process of agglomeration depends on the NPs concentration and size distribution, which are constant for the same NPs generator parameters.

The SERS structures studied in this study were formed from three types of NPs. The first type represents agglomerates of primary NPs, actively exhibiting properties of plasmonic nanoantennas that support longitudinal oscillations in electron density and attenuating radiation in the IR range. The second type is thermally modified NPs obtained by exposing primary NP agglomerates to high temperatures (750 °C) when they pass through a tube furnace. In this process, the agglomerates that pass through the high-temperature area turn into individual spherical NPs exhibiting plasmonic properties. However, during their further transportation in a hot gas, NPs agglomerated for the second time due to their more intense Brownian motion at high temperatures, increasing the probability of particle collisions, as the NPs diffusion coefficient is proportional to the environment temperature [26]. The third type is laser-modified NPs obtained by exposing the aerosol flow to pulsed laser radiation with a wavelength of 1053 nm. The interaction of radiation with aerosol NPs was carried out in a cell developed by us previously [26], which combines aerosol flow with a laser beam along its length. In the cell, NPs absorb a part of radiation energy, which is converted into heat and spent on NPs’ partial or complete sintering, practically without heating the environment. In this approach, the best modification results are expected when the aerosol is exposed to pulsed radiation with a pulse duration of fewer than 50 ns because, in this case, the NP sintering process is adiabatic [26,28]. We did not observe strong secondary agglomeration during NP laser modification in an aerosol flow. This occurred due to the low ambient temperatures compared with the thermal modification temperatures in a hot gas stream and the corresponding low Brownian motion velocities of the particles.

In our experiments, we used a pulsed laser with a wavelength of 1053 nm and pulse duration of around 10 ns (TECH-1053, “Laser-export” Co. Ltd., Moscow, Russia). The laser modification process was performed at a pulse repetition rate of 500 Hz and two pulse energy densities: 12 mJ/cm^2^ (maximum) and 4 mJ/cm^2^. Previously, we showed that laser modification at the maximum energy density leaves no agglomerates of primary Au NPs, i.e., a complete modification of Au agglomerates was observed [26].

**Figure 1:**
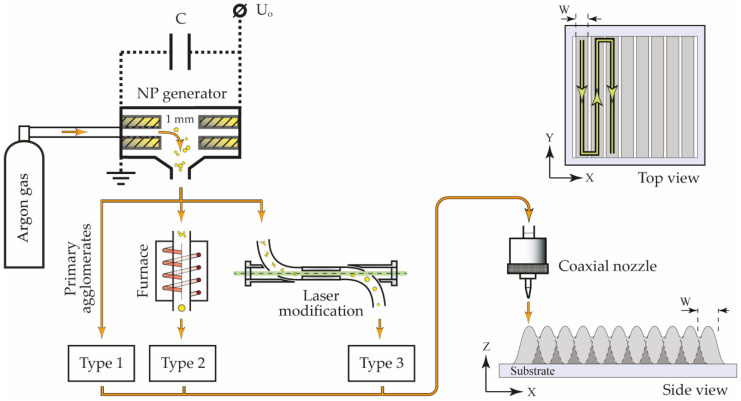
The scheme of the experimental setup with the SERS array printing pattern.

A scheme of the aerosol printer is shown in Figure 1. Aerosol NPs were deposited through a coaxial nozzle with a 300 µm outlet, which determined the width of the printed line W ≈ 300 µm and was located at a 2 mm distance from the substrate. NP arrays were printed on substrates placed on a movable computer-controlled coordinate table according to the pattern shown in Figure 1 by the green line.

The NPs morphology was characterized with a scanning electron microscope (SEM) JSM 7001F (JEOL, Ltd., Tokyo, Japan). SEM images were also used to determine the NPs’ surface density, the average size of modified NPs, and the percentage of individual NPs in the array by statistically processing 400 pieces on average.

### 2.2. Analytes Deposition

**Figure 2:**
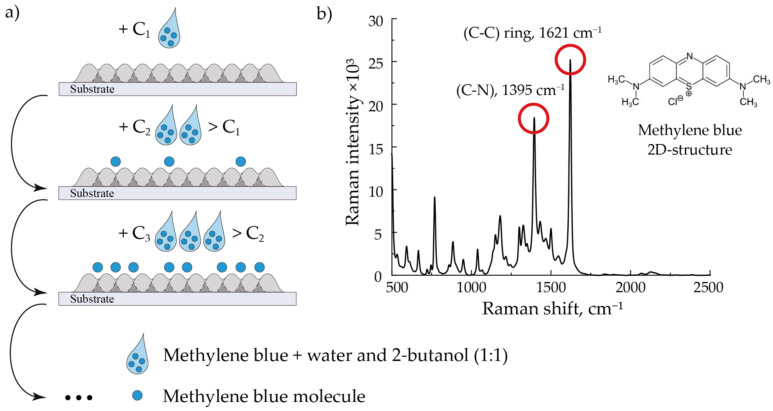
The sequential method of applying an analyte to an array (**a**) and the characteristic MB SERS spectrum (**b**). The spectrum corresponds to a MB 10^−4^ M solution deposited on an array of Au NPs modified by laser radiation at a flow rate of 400 mL/min.

In this work, we studied the printed SERS structures’ ability to amplify the Raman signal. MB crystals dissolved in a mixture of distilled water and 2-butanol in a ratio of 1:1 at concentrations 10^−3^, 10^−4^, 10^−5^, and 10^−6^ M were used as an analyzed substance. For studying the SERS signal of MB at these concentrations, we applied 1 μL of its solution over the studied arrays of NPs layer-by-layer by a pipette dispenser (Figure 2). First, the SERS signal was studied for the MB solution with a minimum concentration of 10^−6^ M applied to the arrays of NPs under study. Next, solutions with concentrations of 10^−5^, 10^−4^, and 10^−3^ M were similarly applied to the structures for sequential study of their SERS signal. After each application, we left the substrates for 10 min for their complete drying, which led to the formation of an analyte solid phase distributed over the structure. An example of the MB SERS spectrum is shown in Figure 2. In these experiments, we also determined the spontaneous Raman signal to calculate the enhancement efficiency of the fabricated substrates. For this purpose, we determined the Raman spectrum for 1 μL of 10^−3^ M MB solution applied over an aluminum oxide substrate with a pipette dispenser.

### 2.3. Spectra Measurements

The absorption spectra of printed structures made of NPs of three types on quartz glasses were measured with a spectrophotometer V-770 UV-Visible/NIR (JASCO, Easton, MD, USA) in the wavelength range of 200–1100 nm.

The Raman spectra were studied on a DXR Raman microscope (Thermo Fisher, Waltham, MA, USA), with a CW laser excitation at a wavelength of 780 nm, with an average power controlled in the range of up to 24 mW. Measurements were recorded with a 10× objective, which averaged the backscattered signal over a large area of the structure, covering more than 1000 particles at once by a beam with a diameter of 30 μm. This ensured the independence of the signal from the particles’ stacking and orientation on the substrate. It also minimized the energy density of the laser radiation and reduced the destruction of the MB chemical bonds, which slowed down the degradation of the signal during measurements. To achieve the best signal amplification and signal-to-noise ratio, the optimal power of the laser source, aperture diameter, exposure time, and number spectrum for their averaging from one area were chosen to be 5 mW, 50 μm, 2 s, and 5 shots, respectively. To reduce the influence of the deposition inhomogeneity on the SERS structure, the spectra were additionally averaged from 5 randomly selected areas.

The signal enhancement efficiency was determined by the two most intense peaks characteristic of MB, 1395 cm^−1^ and 1621 cm^−1^, corresponding to (C–N) and (C–C) vibrational bonds of the analyzed molecule, respectively (Figure 2 (on the right)) [29].

## 3. Results and Discussion

### 3.1. Structural Properties

**Figure 3:**
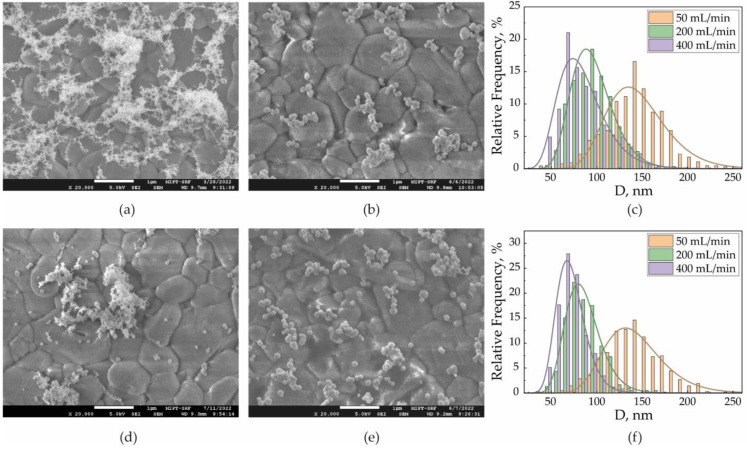
SEM images of SERS arrays fabricated with the aerosol flow of 50 mL/min from (**a**) primary agglomerates, (**b**) thermally modified NPs, and (**d**) laser-modified NPs with 4 mJ/cm^2^ and (**e**) 12 mJ/cm^2^ energy density. Size distributions for (**c**) thermally modified NPs and (**f**) laser-modified NPs with 12 mJ/cm^2^ energy density.

The arrays formed from the three types of NPs fundamentally differed in the degree of their agglomeration. In the first type, all NPs were agglomerated. In arrays of the second and third types, the agglomeration of NPs decreased in the following order: laser-modified NPs with an energy density of 4 mJ/cm^2^, thermally modified NPs, and laser-modified NPs with an energy density of 12 mJ/cm^2^. Characteristic SEM images that brightly demonstrate the differences between the studied types of arrays are presented in Figure 3a,b,d,e for a gas flow rate of 50 mL/min.

First, it was of interest to compare two methods of NPs’ complete modification by high temperature and by laser radiation with an energy density of 12 mJ/cm^2^. The surface density of NPs in the arrays was in the range of 8–10%. Because the initial agglomerates were completely modified in both cases, the sizes of individual aerosol NPs were close for the same gas flows. Thus, at the gas flows 50, 200, and 400 mL/min the average sizes of the aerosol NPs were d_n_ = 135, 80, and 60 nm, respectively (Figure 3c,f). However, the SEM images show that the second type of array contained more secondary than the third type (Figure 3b,e). Thus, by directly counting the number of individual NPs in the SEM images, we determined the percentage of individual NPs out of the number of all separately deposited particles. Initially, this fraction depended on the concentration and mobility of NPs in the flow, which together determined the NPs’ agglomeration tendency and were identical for the same type of array formed with the same flow rates. Here, for the third and second types of arrays, we found that the percentage of individual NPs in each array did not decrease with an increase in the gas flow, but the difference decreased (Table 1).

### 3.2. Optical Spectra Characterization

**Figure 4:**
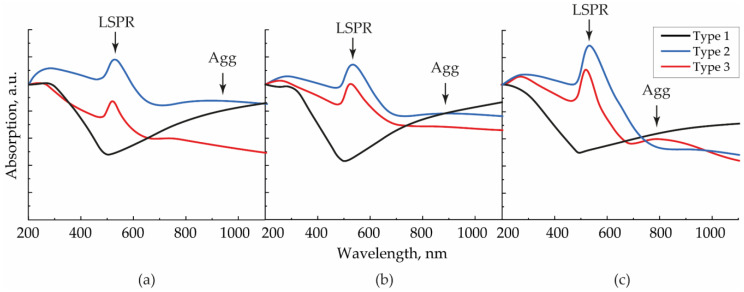
Absorption spectra for SERS structures of types 1–3, formed with aerosol flows (**a**) 50, (**b**) 200, and (**c**) 400 mL/min.

The ratio of the number of individual NPs and secondary agglomerates was confirmed by the absorption spectra of similar arrays fabricated on quartz glasses (Figure 4). It is well known that the peak at the wavelength *λ*_LSPR_ = 528 nm, which we observed for the second and third types of arrays and was absent in the first type, corresponds to the excitation of localized surface plasmon resonance (LSPR). On the contrary, a rise in the IR region absorption is a result of the LSPR redshift due to an increase in the size of the oscillatory system and corresponds to longitudinal oscillations on NP agglomerates [26,30]. This type of oscillation was evident for arrays of the first type (black curve). The redshift Δ*λ* for contacting modified NPs with a diameter *d_p_* can be described by the empirical formula in the approximation of the absence of quantum tunneling in the contact zone [31]:

(1) $$\Delta \lambda =0.004\cdot \lambda_{\mathrm{LSPR}}{\left(d_{p}\right)}^{1.1}$$

Taking into account the experimentally measured average NP diameters at the gas flow rates of 50, 200, and 400 mL/min, the contour corresponding to longitudinal vibrations on individual agglomerates, according to Equation (1), could be detected at around 1000, 800, and 720 nm, respectively. In the example of second and third type arrays, the maxima were found at different wavelengths (940, 880, and 800 nm) in this sequence because, in reality, the spectral position depends on the number of particles in the considered agglomerate and its configuration. In this case, the width of the resonant contour of longitudinal vibrations and, consequently, the absorption value, were determined by a large variety of sizes of randomly oriented agglomerates on the substrate. Moreover, in the absorption spectra of the structures, one may notice Fano resonances, causing a slight shift in the interacting peaks of LSPR and longitudinal vibrations on agglomerates. It is essential to highlight the absence of the peak at 800 nm for the second type structure, deposited with the flow rate of 400 mL/min, and its presence for the third type structure. Because the difference between NPs’ fractions of these structures was the smallest (Table 1), their spectra should have demonstrated close resonances. However, due to the destructive interference between the broader LSPR and longitudinal vibrations on agglomerates for the second type structure, the peak at 800 nm disappeared.

According to the absorption decrease in the IR region with the rise in the gas flow, we concluded that the agglomeration degree for NPs in the second type arrays decreased and approached the agglomeration degree of the third type array, which remained approximately constant at all gas flow rates.

### 3.3. Raman Spectra Characterization

**Figure 5:**
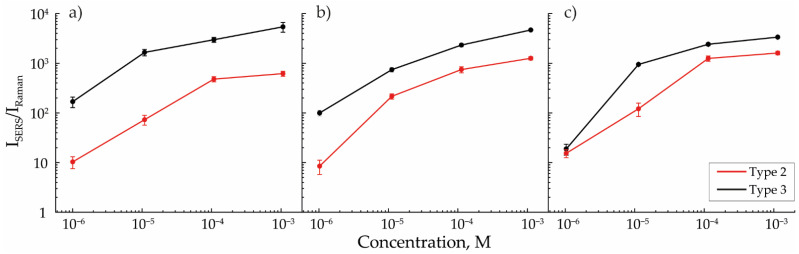
Efficiency of MB detection for SERS arrays of types 2 and 3, formed with aerosol flows of (**a**) 50, (**b**) 200, and (**c**) 400 mL/min.

The enhancement in the Raman signal after MB was applied over the studied structures strictly correlated with the ratio of the number of individual NPs and agglomerates in an array. Figure 5a–c shows the dependencies of the intensity of the MB SERS signal I_SERS_ measured on the surface of the structure, reduced to the intensity of the MB signal I_Raman_ measured on aluminum oxide. This value may be interpreted as the detection efficiency of the deposited substance. In this study, to reduce the error, the I_SERS_/I_Raman_ values were averaged over the two peaks characteristic of MB (Figure 2b) from the different concentration spectra averaged from five randomly selected areas on the samples. This ensured that the error was much lower than the measured value and lower than the difference between the values corresponding to the studied types of structures (less than 10% in the worst cases). The reproducibility of the result was ensured by the independency of the studied signal from the form and orientation of agglomerates as the main studied signal emitted from the structure’s “hot spots” was approximately the same for all particle morphologies in one type of structure. Comparable values of detection efficiencies for arrays of NPs modified with a laser with 12 mJ/cm^2^ energy density were obtained in other studies [32,33,34].

Raman signal enhancement was higher for arrays of NPs modified by laser radiation with an energy density of 12 mJ/cm^2^ for all cases of aerosol flow rates. However, with the increase in the flow rate, the detection efficiencies for the second and third types of arrays converged, which was explained by the high similarity between these arrays in terms of the ratio of the number of individual NPs and agglomerates in their composition. This peculiarity, in turn, was explained by the absence of secondary agglomeration effects for the third type of array and by a decrease in secondary agglomeration with the rise in the gas flow rates for the second type of array, which resulted in the increase in the detected signal intensity. However, as the flow rate increased, the average size of aerosol NPs decreased, and the efficiency of plasmon resonance excitation by the 780 nm laser source also became lower [35,36]. This effect may additionally arise due to a decrease in the coverage density caused by the analyte molecules of individual NPs and their agglomerates. This means that with a linear decrease in the diameter of NPs, the number of analyzed molecules covering the Au surface decreases as a square. Due to these two described factors, the SERS signal’s enhancement decreases with gas flow increase. The higher signal obtained on arrays with a higher number of individual NPs allowed us to predict a lower theoretical limit of MB detection [37], which occurs when the SERS efficiency is compared with the spontaneous Raman scattering efficiency, i.e., when I_SERS_ = I_Raman_. Thus, the theoretical limit for the third type of array, equal to 10^−11^ M, was significantly lower than for the second type, equal to 10^−8^ M.

**Figure 6:**
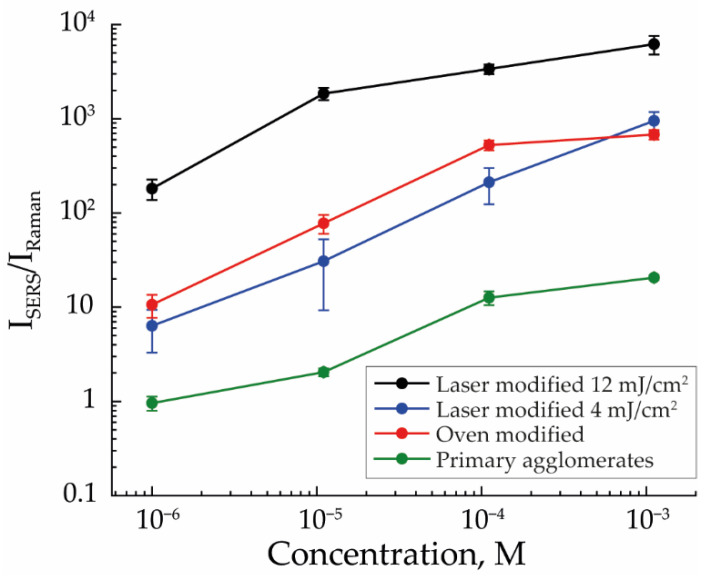
The efficiency of MB detection for SERS structures fabricated from primary agglomerates, thermally modified NPs, and laser-modified NPs with 4 mJ/cm^2^ and 12 mJ/cm^2^ energy density with an aerosol flow rate of 50 mL/min.

To confirm the influence of agglomeration, it was important to compare the detection efficiency of MB on arrays of completely modified NPs with the detection efficiency on arrays consisting of pure agglomerates. For this, arrays were formed from agglomerates of primary NPs and from NPs with incomplete modification by laser radiation (with an energy density of 4 mJ/cm^2^), representing a transitional stage between the first and third types of arrays. Because the structure of these arrays did not change at different flow rates, the arrays formed at a flow rate of 50 mL/min were used to compare the efficiency of MB detection (Figure 6).

The efficiency of MB detection was the worst for the first type of array; at the concentration of 10^−6^ M, it does not exceed the efficiency of spontaneous Raman scattering. However, if we added individual NPs to this type of array, as was realized in the case of arrays of NPs modified with laser radiation with an energy density of 4 mJ/cm^2^, the MB detection efficiency rose to the level of thermally modified NPs’ arrays. As shown earlier, SERS signal amplification is determined by both the ratio of the number of individual NPs in the array and their sizes. However, in the case of arrays with NPs incomplete modification, the balance of these two factors did not allow for a result similar to the third type of array. Presumably, this was a consequence of the low ratio of the number of individual NPs, which was difficult to calculate in these structures due to the impossibility of determining the number of agglomerates. The enhancement effect was also weakened due to the small average size of individual NPs, equal to 83 nm, completely modified from agglomerates for which the laser energy was sufficient.

## 4. Conclusions

In this study, we investigated the Raman signal amplification of SERS structures fabricated by ADP on an example of MB dye. The structures were deposited on aluminum-oxide substrates in the form of square arrays and were made of three types of Au NPs: agglomerates of primary NPs, thermally modified NPs at the temperature of 750 °C, and NPs modified by nanosecond pulsed laser radiation with a wavelength 1053 nm. SERS measurements of MB were performed under the influence of low-power CW laser radiation with a wavelength of 780 nm.

The highest Raman signal enhancement was found for arrays consisting of NPs modified at the maximum laser pulse energy density (12 mJ/cm^2^). The smallest amplification was observed on arrays formed from agglomerates of primary NPs. Arrays of thermally modified NPs and NPs modified by laser radiation under exposure to pulses with an energy density 4 mJ/cm^2^ demonstrated similar intermediate results in amplification. The order may be explained by the ratio of the number of individual NPs and agglomerates in the composition of the studied array. This effect was determined by directly calculating the percentage of individual NPs in the array and was confirmed by the absorption spectra of similar arrays deposited on quartz glasses. The ratio plays an important role in SERS signal enhancement, as individual NPs can demonstrate a stronger field localization than agglomerates under exposure to pump laser radiation with a wavelength of 780 nm. In this regard, complete modification by pulsed laser radiation is better than thermal modification because the former allowed us to obtain a larger number of individual NPs due to the absence of secondary agglomeration effects in the gas stream. On the other hand, the effects of secondary agglomeration can also be reduced for thermal modification by increasing gas flow rate because the time spent by particles in the gas and, consequently, the time allotted for the agglomeration processes is reduced. At the same time, a change in the gas flow rate causes a change in the size of initial agglomerates and, consequently, in the size of modified NPs, which is smaller at high flow rates. This inevitably influences SERS efficiency, which drops down with a decrease in the size of spherical NPs due to the blueshift of LSPR when using a wavelength of 780 nm. To conclude, individual NPs in the array increase the intensity of SERS signal, but the effects of agglomeration of NPs, on the contrary, reduce it. Furthermore, it becomes evident that an increase in the number of individual particles leads to a distance reduction between them, which, together with the parameter of the NPs’ average size, can affect the effects of SERS signal enhancement.

## Figures and Tables

**Table 1:** Individual NPs fractions for SERS arrays of second and third types.

Aerosol Flow, mL/min	Individual NP Fraction, %		Difference, % (Type 3–Type 2)
	**Type 3**	**Type 2**	
50	51 ± 3	22 ± 1	29
200	76 ± 4	65 ± 3	11
400	76 ± 4	68 ± 3	8
